# Practical tips for a fast and successful transition to an online curriculum

**DOI:** 10.12688/mep.19751.2

**Published:** 2024-07-11

**Authors:** Chloé E.C. Bras, Remco C. Jongkind, Ellen L. van Veen, Kim Win Pang, Laura E. Olthof, Tobias B.B. Boerboom

**Affiliations:** 1 Teaching & Learning Centre, Amsterdam University Medical Centres, Amstersdam, North Holland, The Netherlands; 2 Faculty of Veterinary Medicine, Utrecht University, Utrecht, Utrecht, The Netherlands

**Keywords:** online education, community of inquiry model, curriculum transition

## Abstract

The COVID-19 pandemic and the following lockdown forced educational institutions to transform their face-to-face curriculum into an online programme in a matter of weeks. In this article, we present 12 tips for a successful transition based on the challenges that we faced in the Bachelor of Medicine at Amsterdam Medical Centre. These tips are divided in four main themes: infrastructure, faculty development, student engagement, and teaching activities. The Community of Inquiry model is used as backbone in all tips, since the three elements, teaching presence, social presence, and cognitive presence are essential factors in effective online education. These tips can be useful for everyone who wants to implement online education in their curriculum, whether borne out of necessity or by design.

## Introduction

In early 2020 the COVID-19 pandemic turned the world upside down. Suddenly, education could no longer take place face-to-face and entire programmes turned into online curricula in a matter of weeks. On the level of students, lecturers and content, challenges arose in knowledge and skills ( Kebritchi *et al.*, 2017). However, with technology evolving fast, online or hybrid education has the potential to be effective, easily accessible and sustainable ( Protopsaltis & Baum, 2019). The educational institute plays a major role in facilitation of the transition to online education, however they need to know what challenges they are facing and what possible solutions there are for effective online programmes ( Kebritchi *et al.*, 2017).

Simply converting traditional courses into an online version neglects the complexity of education. A more broad framework for effective online educational programmes is described by Richardson *et al.* (2012) in the Community of Inquiry (CoI) model. This framework is based on the framework of Garrison *et al.* (1999). They state that the quality of online education depends on the relation between three key aspects: social presence, teaching presence, and cognitive presence. Social presence is described as the student-student interaction, and the extent in which they feel safe and involved in the online environment. It comprises of affective expression, open communication, and group cohesion. Teaching presence refers to the factors of effective online education that can be influenced by lecturers and the educational institute. Three key components of teaching presence are instructional design and organization, facilitating discourse, and direct instruction. Cognitive presence is the students learning process, which consists of four phases, i.e. triggering, exploration, integration, and resolution ( Richardson *et al.*, 2012). A triggering event leads to exploration of the possibilities. Due to the exploration, new insight can be integrated in already acquired knowledge and skills, which can eventually lead to resolution.

**Figure 1.**
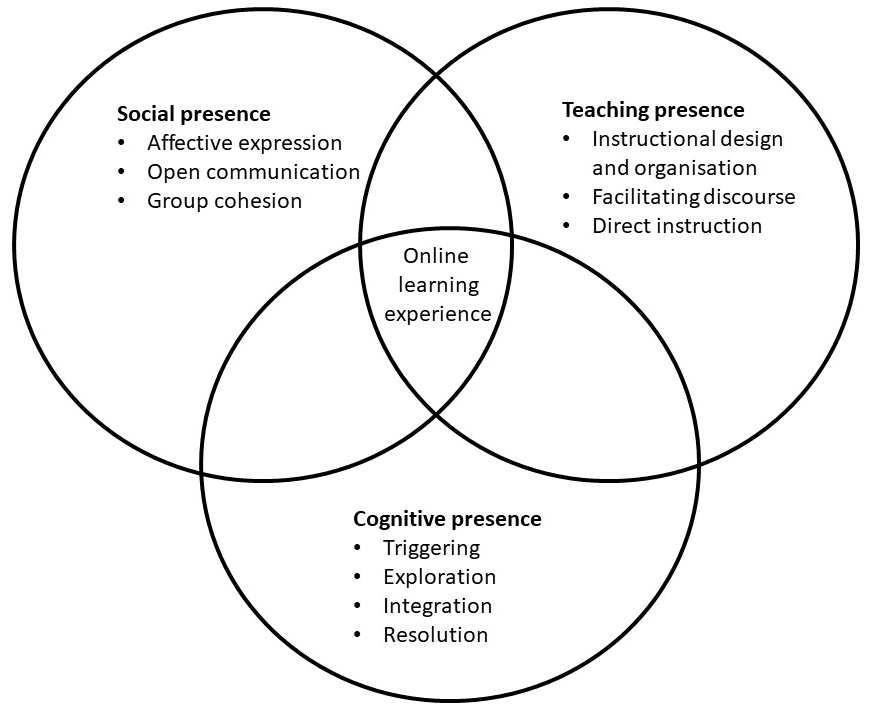
Community of Inquiry Framework for online learning experience. The Community of Inquiry model described by ( Richardson *et al.*, 2012) states that the quality of online education depends on the relation between three key aspects: social presence, teaching presence and cognitive presence. Social presence is described as the student-student interaction, and the extent in which they feel safe and involved in the online environment. Teaching presence refers to the factors of effective online education that can be influenced by lecturers and the educational institute. Cognitive presence is the students learning process. This figure has been reproduced from the Community of Inquiry website with permission from Richardson *et al.* (2012). It is adapted from learning experience to online learning experience, and the three elements are further elaborated upon.

In the current article, the Community of Inquiry model (see Figure 1) is used as a framework to provide guidance in developing effective online programmes. We present 12 tips to digitalise a curriculum, in which continuity and quality are maintained. These tips are divided in four main themes, i.e., infrastructure, faculty development, student engagement, and teaching activities. It is based on our experiences in the Bachelor of Medicine at Amsterdam University Medical Centres (UMC), location Amsterdam Medical Centre (AMC) and supported with literature.

### Tip 1: Faculty development - Stimulate collaboration and communication in the core educational team

When transitioning to an online curriculum it is important to facilitate and unite your faculty for optimal teaching presence: get together with a core educational team of people who teach the most in regular meetings and create short lines of communication between management, ICT (information and communications theory), lecturers and support staff to choose the approach and tools to digitize the programme ( Kirk & MacDonald, 2001; Miyagawa & Perdue, 2020). During COVID-19 the transitioning had to be very fast and everyone was on a tight schedule. To keep eachother updated, we started every monday with a meeting which led to cooperation instead of everyone working independently. To keep all lecturers, also outside of the core educational team, up to date on the latest policies, developments and possibilities, weekly mailings were sent or live question sessions were arranged.

Furthermore, to streamline communication ideally pick one person in your core team as a representative within and outside the faculty to allow short lines of communication. This enhances the sharing of best practices, policies, and developments within the faculty and other faculties of the university.

### Tip 2: Infrastructure - Ensure a solid basis of streaming and interaction software and (limit) digital tools

Streaming lectures is a key component of an online curriculum with many benefits if used correctly and under the right conditions ( Mosley, 2017; Wirihana *et al.*, 2017). One must assure that both the hardware and software infrastructure are well arranged to ensure good teaching presence ( Chow & Croxton, 2017).

Regarding the hardware it is important that there are locations with live streaming equipment, such as stable internet connection, good microphone, and camera. These should be accompanied by technical support and a moderator to control the chat and interactive functions such as breakout rooms ( Bordes *et al.*, 2021; Norman, 2017). Planning of time and location of the lectures in the streaming room should be communicated well to lecturers, moderators and students via a centralized schedule. In our institution, we set-up three main rooms with good microphones, a camera and stable internet connection from where we could broadcast live lectures. We hired student assistants, who were trained in moderating the online lectures and helped the lecturers with monitoring the chat and sharing the slides. Furthermore, there was someone responsible for technical support who could be called on in case of problems.

Regarding the software it is important to use a limited number of digital tools to ensure maximal proficiency of the students and lecturers and to make it possible for the ICT department to provide the necessary support ( Margaryan *et al.*, 2011). The absolute minimum that is required is a live streaming tool with options for breakout rooms such as Zoom, Webex, or Teams and ideally one would also have access to an interaction tool such as Mentimeter, Kahoot, or Miro.

### Tip 3: Infrastructure - Guarantee access and proficiency in the use of assessment tools

A house cannot be built without strong foundations: access to hardware and software for lecturers and students needs to be guaranteed. Hence, contact points need to be created where students or lecturers can either borrow laptops or provide spaces to participate in digital learning and testing ( Khalid & Pedersen, 2016).

Furthermore, training sessions should be organised for lecturers and students in order to optimize the continuity and reliability of teaching activities and teaching presence. Since assessment of students is a high-stake element in education, accessibility and functionality of the exam platform is imperative before an examination moment ( Elsalem *et al.*, 2020). We organised a test examination in advance to check the accessibility and functionality and in case of difficulties alternative options were offered.

### Tip 4: Faculty development - Implement formal faculty development initiatives

In a considerable number of medical schools, faculty and lecturers have little experience with conducting their courses entirely online ( Lewis & Baker, 2010). Online teaching requires faculty to adapt their usual teaching materials and methods to facilitate learning and encourage interaction ( Lockyer *et al.*, 2006). As stated in the introduction section, lecturers should consider social presence, teaching presence, and cognitive presence when they teach online. This requires new skills and therefore creates an urgent need for faculty development on this matter ( Albrahim, 2020; Zuo & Miller Juvé, 2021). However, due to COVID-19, organizing faculty development meetings was challenging. On the one hand face-to-face gatherings of staff were restricted in many cases and on the other hand a lot of medical teaching staff faced overwhelming demands of their clinical task.

As a form of individual formal faculty development, we provided lecturers with short one-topic videos and e-learning assignments on online teaching. These videos and assignments covered both online didactics and practical instructions on how to choose and use the selected online teaching tools. Educational redesign aids for online teaching were distributed through mailings and an newly created website. In addition to these forms of individual formal faculty development we organized several online instruction and question and answer (Q&A) sessions for groups of lecturers. Lecturers were also invited for seminars on online Team Based Learning and interaction in online webinars.

### Tip 5: Faculty development - Facilitate informal faculty development initiatives

Besides the abovementioned formal faculty development activities, institutes are also advised to implement several informal initiatives in the workplace to aid lecturers in their transition to online teaching ( Baker, 2020; Steinert *et al.*, 2010). Working alongside more experienced colleagues, and having the opportunity to consult them for feedback and mentoring greatly enhances learning in the workplace ( Eraut, 2007). As stated before, a lot of our medical teaching staff faced overwhelming demands in their clinical tasks, so they didn't had time to professionalize in online teaching. Therefore, as a form of individual informal faculty development we paired our dedicated lecturers (Medical Educators) with significant skills and knowledge concerning online teaching, with colleagues with less experience in online teaching. By pairing Medical Educators with medical content experts, the organization and implementation of online education is enhanced and progressed more rapidly, leading to better teaching presence.

To enhance interaction between these two groups of lecturers, the Medical Educators took the role of moderators in the majority of online seminars, Team Based Learning sessions and tutorials. This created lots of opportunities for role modelling, consulting and feedback in the online workplace. In a later phase, student assistants who were trained by the Medical Educators also became supporting moderators.

To further enhance informal faculty development in online teaching, we organized weekly online teaching meetings where lecturers could exchange experiences with their online courses and where they could ask for peer support.

### Tip 6: Student engagement - Avail yourself of student feedback

Student feedback regarding online education provides critical knowledge and understanding for lecturers. Students' perspective on educational design and organisation, can contribute to better teaching presence. Moreover, it can lead to improved student engagement in educational activities once they recognize the importance of feedback ( Brody & Santos, 2019; Whittle *et al.*, 2020).

We used two types of student feedback in our digital curriculum: focus groups and regular online questionnaires. By setting up a student focus group, we made sure that educational goals and expectations of students were aligned. Regular meetings with this group gave students the opportunity to make their voices heard. Furthermore, after lectures a digital evaluation form was sent by using a QR-code. Fast repeating evaluation moments made it possible to immediately implement the feedback about online education in our curriculum. We advise to focus on student feedback mostly at the start of the course or when using different types of educational formats, since the risk of ‘evaluation tiredness’ lies ahead when students are asked for feedback during every class or lecture ( Adams & Umbach, 2012).

### Tip 7: Student engagement - Pay attention to students’ mental support

From the abovementioned student panel, we also received signals about sense of isolation and mental burden during the period of online education. Also tutors shared their concerns about the mental wellbeing of their students based on their online individual meetings with students. The impact of quarantine during the COVID-19 pandemic has led to medical students feeling emotionally detached from family, peers and friends leading to a decrease in overall work and study performance ( Meo *et al.*, 2020). Since mental burden has an impact on students’ study behaviour, we put extra emphasis on (continuing) the tutoring and coaching online. Tutors were instructed to pay special attention to the student's mental health and stimulate community feeling in class to improve their social presence.

Furthermore, students receive an overwhelming amount of information during the transition from on-campus education to full online education. To provide an overview we set-up a central page on the learning platform for communication of all changes during the COVID-period. One of the items on the central page on the learning platform was highlighting the route for mental care. The attention for students' mental support is necessary for open communication to improve the social presence.

### Tip 8: Student engagement - Stimulate peer support to prevent isolation

Social presence is an important aspect to promote study success ( Richardson *et al.*, 2012). Lack of social interaction can lead to student isolation, therefore it is important to stimulate peer support during the course ( AlJhani *et al.*, 2022; Croft *et al.*, 2010). However, in an online environment it can be challenging to create social interaction and group cohesion without face-to-face contact. Due to COVID-19 students didn't have a lot of social interaction and as stated in Tip 7 did we receive signals about sense of isolation and mental burden. Therefore, we stimulated peer support inside and outside the classroom to prevent isolation and indirectly promote study success in an online curriculum.

In our online sessions we used active teaching methods in which students worked together in small groups. For example, an assignment was explained to all students in the main room and then everyone went to their breakout room to discuss and formulate answers. After a given period of time, all students came back to the main room to discuss answers led by a teacher. In synchronous teaching activities it is also possible to let advanced medical students serve as teaching assistants. These advanced medical students can help other students to understand the teaching material based on their own experience as a student ( Mesner Fireizen *et al.*, 2023).

To stimulate social interaction besides the online sessions, asynchronous small group-assignments can be implemented in the curriculum ( Bickle & Rucker, 2018). We developed assignments in which students had to work together on a paper and in which they had to provide peer feedback on a presentation as a formative assessment.

Encouraging student initiatives is another way to create a student community in which students can interact and learn with peers ( Richardson *et al.*, 2012). An example of such a student-lecturer initiative is an organized (online) pub quiz with questions about the study material prior to the exam.

In short, peer support can be stimulated by lecturers inside and outside classroom activities as well as by student initiatives. 

### Tip 9: Teaching activities - Create a safe and supportive online learning environment

Building an online learning community in which students feel safe and supported is crucial to facilitate learning ( Sun & Chen, 2016). Moreover, a sense of belonging, open communication and expression of emotion can prevent students from dropping out ( DiRamio & Wolverton, 2006; Garrison *et al.*, 1999). In our online teaching sessions, the teacher was continuously visible to make the lecture more personal. Students were welcomed at the start, and during the session the teacher used first names to make students feel part of the community.

Since team-based learning is a key element in our curriculum, small-scale education takes place in fixed groups of twelve students, which ensures strong social presence. A disadvantage of these fixed groups in online education is logistics like grouping students in the right online session and a fluctuating attendance. Alternating groups results in less logistic difficulties and stimulates students to collaborate with various fellow students. However, it reduces the social presence. In our experience, fixed groups are important in the first year to provide structure and a safe and supportive learning environment. Whereas third year students adapted faster to a new group.

Besides social presence, teaching presence is an important factor to create a supportive learning environment ( Yuan & Kim, 2014). Especially in online education, lecturers are not only content experts, but they are also facilitators of interaction ( Richardson *et al.*, 2012). When we started with online education a lot of students turned off the camera and some changed their name in a stripe, so their names could not be mentioned. To ensure a safe learning environment, we made a code of conduct which was implemented in all online sessions. As a result, more students turned their camera on and all students could be mentioned by their name. When there is more time to create an online learning environment, it is recommended to write the code of conduct together with the students, so they take ownership and responsibility for the codes ( Coleman, 2012).

### Tip 10: Teaching activities - facilitate interaction, dialogue and student participation

We consider interaction to be one of the key components in the design of our curriculum. By triggering students and facilitate their exploration process, cognitive presence is stimulated ( Richardson *et al.*, 2012). Students value to be in constant dialogue with their lecturers ( Boelens *et al.*, 2017), and student-teacher interaction as well as peer interaction is important for social and teaching presence, leading to effective learning ( Delgaty, 2018). In an online learning environment, it can be challenging to facilitate sufficient interaction with fellow students and lecturers ( Croft *et al.*, 2010). During the online lectures we saw students sitting at home and get easily distracted, so we intended to maintain interaction in the design of our online curriculum in the following ways;

We communicated dates and times of all live online sessions at the beginning of each course and at the start of every week. During live classes, in small or larger groups, we frequently built-in moments for questions. With large student groups, the chat or Q&A function of the video conferencing platform could be used by students to meet this purpose. Ideally, the chat was managed by a second teacher/moderator. In live sessions with larger groups of students, interactive voting tools (e.g., polls in Zoom and Mentimeter) proved to be useful in creating interaction. Results of the voting tool could serve as a base for plenary discussion, and student engagement could be encouraged by asking them to motivate their choices.

Peer interaction was be facilitated by creating small online subgroups (breakout rooms). We gave students a specific and comprehensible assignment to carry out in a group and provided feedback afterwards in the main session. Furthermore, weekly online (live) Q&A sessions were scheduled. Students were asked to hand in questions beforehand, so lecturers could provide additional explanation where needed. We observed better attendance to these online gatherings, as opposed to face-to-face question hours. 

Ultimately, we would like to emphasize lecturer's availability outside of live moments. Asynchronous discussion boards could be a useful medium for peers to collaborate, provided that lecturers take an active role in supervising the discussions and providing guidance. 

### Tip 11: Teaching activities - Be creative and variate in teaching methods

Because students all have a preferred learning strategy, variation in teaching methods is just as important as interaction to increase motivation and the effectiveness of student learning ( Zayapragassarazan, 2020). The way an educator presents information may stimulate and facilitate one student’s learning but could impede the learning process of another student. We enhanced the cognitive presence by creating variation in teaching and learning activities in our programme to trigger students in different ways and to align with as many learning strategies as possible ( Richardson *et al.*, 2012).

Many different teaching methods are suitable to create this variation, even in a digital or hybrid curriculum. For example, we used breakout rooms, to make them work together in small groups on a project, such as creating a differential diagnosis or assessing an electrocardiogram. Afterwards, we discussed the results in a main session together and gave the students feedback. Furthermore, we used gamification ( Boelens *et al.*, 2017) to walk them through taking a patient history, by simultaneously playing an online game together with students, using share screen options.

In our experience, a lot of teaching activities can be digitalized by creatively using existing content and educational formats. Some classes, like radiology and microbiology, turned out to be very suitable for online education and are even better received in a digital environment. During the class students indicated that they were able to see all radiology images in high quality with the good light and that they would like to receive these tutorials in a digital environment from now on.

Admittedly, not every teaching activity can be digitalized. Practicing specific practical skills is not possible to digitalize, however anatomy tutorials with a video or photo images from specific structures proved to be useful in the explanation. Students have a better view on the heart when watching an anatomy tutorial than when gathering around a table to see which small structure the lecturer pointed out. So in that case, online education can be a valuable addition to face-to-face education ( Bordes *et al.*, 2021).

### Tip 12: Teaching activities - Take cognitive load into account when scheduling online sessions

Laptops and mobile phones are known distracters during class, which can lead to cognitive overload ( Attia *et al.*, 2017). Moreover, we experienced that it is more difficult to sense students’ energy level in the online sessions. Therefore, the design and organization aspect of teaching presence plays an important role to prevent from cognitive overload. Cognitive load should be taken into account when scheduling online sessions and planning the student activities.

A balance between synchronous learning activities and self-study needs to be found during online courses. Our student evaluations showed that at least two or three online live sessions per week are desired for interaction. We set a maximum of two hours per online live session with a break halfway of at least fifteen minutes. The attention span of students is approximately 10–15 minutes ( Pearl & Arunfred, 2019), so it is important to divide a session in short parts of various teaching activities. Alternating explanation with interaction can extend students attention span ( Geri *et al.*, 2017).

Besides live lectures or tutorials, we developed short educational videos (max 15 minutes) which covered one specific topic. Teaching presence encourages cognitive presence ( Hosler & Arend, 2012), so by using these short educational videos students have more autonomy in scheduling their learning activities.

## Conclusion

The change from a face-to-face curriculum to a nearly complete online curriculum in early 2020 was born out of necessity. However, over the past year we have also learned that there can definitely be positive aspects to online education in certain situations.

Therefore, in this article we have outlined 12 tips to help others that want to transform their face-to-face education to an (partially) online format in the future. These tips range from how to set-up your infrastructure and faculty development to engaging students and advice for online educational activities.

## Data Availability

No data are associated with this article.
