# Supplementary material for: Practical tips for a fast and successful transition to an online curriculum
Source: MedEdPublish (2016). 2024 Jul 11;13:211. Originally published 2023 Oct 11. [Version 2] doi: 10.12688/mep.19751.2 (PMC11316167; doi:10.12688/mep.19751.2)
Supplement: Peer review history

# Reviewer report: Reviewer response for version 2

**DOI:** 10.21956/mep.21978.r38293

**Published:** 2024-08-23

**Authors:** William S Brooks

**Affiliations:** 1 The University of Alabama at Birmingham, Birmingham, Alabama, USA

This article provides helpful tips to the medical educator intent on converting curricular elements to a fully online format. The following are observations and suggestions to improve the manuscript.

- The introduction provides Community of Inquiry as a framework for the 12 tips, but it was not inherently clear how CoI influenced each of the 12 tips. More explicit links between each tip and CoI would be helpful.
- How were the themes (faculty development, student engagement, infrastructure) decided upon and how do they relate to Figure 1?
- Tip 1 - overlap with tips 4 and 5. Tip 1 felt more general and less practical.
- Tip 3 - needs to focus more on remote assessment. How to ensure exam integrity, academic integrity, proctoring logistics, synchronous vs. asynchronous testing, etc.
- Tips 7 and 8 had too much overlap regarding student mental health. Consider focusing one tip on ensuring student mental health and another tip on building peer camaraderie

If evidence from practice is presented, are all the underlying source data available to ensure full reproducibility?

Yes

Is the topic of the practical tips discussed accurately in the context of the current literature

Yes

Are all factual statements correct and adequately supported by citations?

Yes

Are the conclusions drawn balanced and justified on the basis of the presented arguments?

Yes

Are arguments sufficiently supported by evidence from the published literature and/or the authors’ practice?

Yes

Reviewer Expertise:

Anatomy education, disability education, pedagogy

I confirm that I have read this submission and believe that I have an appropriate level of expertise to confirm that it is of an acceptable scientific standard, however I have significant reservations, as outlined above.

# Reviewer report: Reviewer response for version 2

**DOI:** 10.21956/mep.21978.r38032

**Published:** 2024-08-09

**Authors:** Ahmed Msherghi

**Affiliations:** 1 The University of Texas MD Anderson Cancer Center, Houston, Texas, USA; 2 University of Tripoli, Tripoli, Tripoli District, Libya

This study presents an integrative plan designed to effectively implement online learning within educational institutions and schools. The proposed framework is commendable, as it highlights the importance of a structured approach to online education.

 However, upon reviewing the study, I found myself eager for more detailed insights into this plan's practical applications. Specifically, I was looking for comprehensive tools and strategies that would empower educators to conduct remote sessions seamlessly from home. While the concept of online learning is increasingly relevant, the lack of concrete resources to facilitate effective delivery can hinder its successful adoption.

 Did you develop a specific strategy for addressing these challenges based on what you face in your institution?

 Additionally, based on existing literature, what practical inferences can you draw regarding educators' remote session provision? These insights would greatly enhance the comprehensiveness of your plan and provide much-needed guidance to teachers navigating the complexities of online instruction.

 A deeper exploration of tools, technologies, and pedagogical approaches suited to remote teaching could significantly enrich your study and equip educators with actionable solutions to foster effective online learning environments.

If evidence from practice is presented, are all the underlying source data available to ensure full reproducibility?

Partly

Is the topic of the practical tips discussed accurately in the context of the current literature

Yes

Are all factual statements correct and adequately supported by citations?

Yes

Are the conclusions drawn balanced and justified on the basis of the presented arguments?

Yes

Are arguments sufficiently supported by evidence from the published literature and/or the authors’ practice?

Yes

Reviewer Expertise:

NA

I confirm that I have read this submission and believe that I have an appropriate level of expertise to confirm that it is of an acceptable scientific standard.

# Reviewer report: Reviewer response for version 2

**DOI:** 10.21956/mep.21978.r37913

**Published:** 2024-08-07

**Authors:** Anita Samuel

**Affiliations:** 1 Center for Health Professions Education, School of Medicine, Uniformed Services University of Health Sciences, Bethesda, MD, USA

Thank you for addressing the comments I raised earlier.

 My only lingering concern is how COI figure in this. You note that you have added this information to each tip but I'm not able to see it.

If evidence from practice is presented, are all the underlying source data available to ensure full reproducibility?

Not applicable

Is the topic of the practical tips discussed accurately in the context of the current literature

Yes

Are all factual statements correct and adequately supported by citations?

Yes

Are the conclusions drawn balanced and justified on the basis of the presented arguments?

Partly

Are arguments sufficiently supported by evidence from the published literature and/or the authors’ practice?

Yes

Reviewer Expertise:

Online education, adult education, educational technology, faculty development

I confirm that I have read this submission and believe that I have an appropriate level of expertise to confirm that it is of an acceptable scientific standard.

# Reviewer report: Reviewer response for version 1

**DOI:** 10.21956/mep.21165.r35037

**Published:** 2023-11-23

**Authors:** Assaf Marom

**Affiliations:** 1 Technion Israel Institute of Technology, Haifa, Haifa District, Israel

In this perspective the authors describe how to effectively transform traditional education into an online curriculum. According to the introduction, the authors base their insight on their own experience and formulate it in the form of 12 recommendations, based on the CoI model (Richardson et al., 2012). These recommendations could be very helpful for cases in which an online curriculum is required. 

 Major comments

1. Reading about the authors' personal experiences that led to each of the recommendations would provide valuable insight. What obstacles did you encounter in each category that informed the advice you are providing? For instance, in Tip 5, could you elaborate on the specific events and discussions among the pedagogic team that led to the conclusion that pairing colleagues would be beneficial? 
2. Similarly, could you provide insights into how the implementation of these recommendations within your institute improved the transition to an online curriculum? For instance, in Tip 7, how did you collect information from your students on mental burden? 
3. There is a substantial body of literature that has emerged post-Covid-19, analyzing academic institutions' experiences in dealing with the challenges posed by the crisis. It would be valuable to incorporate discussions of how these recommendations align with or have evolved in response to the insights provided by the post-Covid-19 literature. 

Minor comments

1. I think that a succinct explanation of what the CoI means is required in the abstract. Alternatively, the authors can rephrase the sentence about CoI in the abstract to make clear that what they list are three components of the model, i.e., teaching presence, social presence, and cognitive presence. 
2. ICT (see Tips 1 and 2) should be defined the first time it is used. 
3. The authors' recommendation regarding the stimulation of peer support (Tip 8, paragraph on "stimulating student initiatives") matches our experience during the Covid-19 crisis. In particular, our experience in teaching anatomy during the crisis demonstrates that initiatives from students who act as dissection instructors played a key role in coping with the challenges of the transition to online teaching (Mesner Fireizen et al., 2023). 

Refs. 

 Mesner Fireizen S, Finkelstein A, Tsybulsky D, Yakov G, and Marom A. 2023. Israeli medical faculties' response to the Covid-19 crisis vis-a-vis anatomical education: The cultivation of future academic leadership-A national mixed-methods study. Anatomical Sciences Education 00:1-16.

If evidence from practice is presented, are all the underlying source data available to ensure full reproducibility?

Not applicable

Is the topic of the practical tips discussed accurately in the context of the current literature

Partly

Are all factual statements correct and adequately supported by citations?

Yes

Are the conclusions drawn balanced and justified on the basis of the presented arguments?

Yes

Are arguments sufficiently supported by evidence from the published literature and/or the authors’ practice?

Partly

Reviewer Expertise:

anatomy, human evolution

I confirm that I have read this submission and believe that I have an appropriate level of expertise to confirm that it is of an acceptable scientific standard, however I have significant reservations, as outlined above.

## References

[1] : Israeli medical faculties' response to the COVID-19 crisis vis-à-vis anatomical education: The cultivation of future academic leadership-A national mixed-methods study. *Anat Sci Educ* .2023;16(6) : 10.1002/ase.2310 1158-1173 10.1002/ase.2310 37366773

# Response

**Published:** 2024-07-03

**Authors:** Chloé Bras

**Affiliations:** FdG, Universiteit van Amsterdam, Amsterdam, North Holland, The Netherlands

Dear Assaf Marom,  Thank you for reading and for your feedback. We have made changes to the paper and its uploaded as Version 2. We believe that version to is much better thanks to your review and feedback.  Below we indicate point by point how we have adjusted it in version 2: 

*Reading about the authors' personal experiences that led to each of the recommendations would provide valuable insight. What obstacles did you encounter in each category that informed the advice you are providing? For instance, in Tip 5, could you elaborate on the specific events and discussions among the pedagogic team that led to the conclusion that pairing colleagues would be beneficial?*

- We added some of our experiences and recommendations in all tips.

*Similarly, could you provide insights into how the implementation of these recommendations within your institute improved the transition to an online curriculum? For instance, in Tip 7, how did you collect information from your students on mental burden?*

- When possible we provided insights into how the implementation of these recommendations improved the transition to an online curriculum.

*There is a substantial body of literature that has emerged post-Covid-19, analyzing academic institutions' experiences in dealing with the challenges posed by the crisis. It would be valuable to incorporate discussions of how these recommendations align with or have evolved in response to the insights provided by the post-Covid-19 literature. *

- We added a number of references post-covid-19 to support our arguments.

*I think that a succinct explanation of what the CoI means is required in the abstract. Alternatively, the authors can rephrase the sentence about CoI in the abstract to make clear that what they list are three components of the model, i.e., teaching presence, social presence, and cognitive presence. *

- Good point, we rephrased that sentence.

*ICT (see Tips 1 and 2) should be defined the first time it is used. *

- We gave the full text the first time the abbreviation was used.

*The authors' recommendation regarding the stimulation of peer support (Tip 8, paragraph on "stimulating student initiatives") matches our experience during the Covid-19 crisis. In particular, our experience in teaching anatomy during the crisis demonstrates that initiatives from students who act as dissection instructors played a key role in coping with the challenges of the transition to online teaching (Mesner Fireizen et al., 2023).*

- Thank you for referring to this interesting article. We referred to it in our practical tips.

# Reviewer report: Reviewer response for version 1

**DOI:** 10.21956/mep.21165.r35040

**Published:** 2023-10-20

**Authors:** Anita Samuel

**Affiliations:** 1 Center for Health Professions Education, School of Medicine, Uniformed Services University of Health Sciences, Bethesda, MD, USA

In this 12 tips article, the authors use the Community of Inquiry (CoI) framework to describe a programmatic transition to the online environment. While the CoI framework has been extensively used and studied, it’s application at a programmatic level is novel.

 The points noted below are provided to help strengthen this manuscript:

 Introduction:

 In your Introduction, you say, *The educational institute plays a major role in facilitation of the process. *What ‘process’ are you referring to here? It is not clearly identified in the preceding sentences.

 The Community of Inquiry framework was proposed and developed by Garrison and Archer in 1999. While Richardson et al. have used the framework, Garrison and Archer should be acknowledged.

 You note that your tips are divided into 4 themes. It would be helpful to identify which theme aligns with which tip.

 Furthermore, how do these themes and tips align with the CoI model? This needs to be clearly stated.

 Tip 1 refers to a core educational team. It would be helpful to define what you mean by this. While the title mentions ‘collaboration,’ the tip itself primarily discusses communication. Clarification is needed here.

 The title for Tip 2 refers to educational ICT infrastructure. However, you only discuss streaming technologies. Educational ICT is much broader than this. You might want to consider reframing your title for this tip.

 In paragraph 2 of Tip 3, you say “abovementioned tools.” What tools are you referring to?

 Similar to tip 2, there is a disconnect between the title and description of Tip 3. The title of Tip 3 refers to digital tools. But the only tools you really discuss are assessment tools. While assessment tools can fall under digital tools, they are not the ONLY digital tools.

 Tip 4: In paragraph 2, you say, *To enable everyone involved in online teaching to participate in faculty development activities, both formal and informal faculty development approaches should be used. *Since the title for the tip mentions ‘formal’ initiatives, this sentence can be removed since the next tip is about informal initiatives.

 Tip 7: Paragraph 1 uses the phrase, *during the same period*. What period are you referring to here?

 Tip 8: The word “stimulate” is used multiple times. It might help to use different words.

 Tip 9: Paragraph 1 you use the phrase “the teacher streamed with video and audio.” I’m not sure what you mean here. Some clarification on what you mean by “streamed with video and audio” would be helpful.

If evidence from practice is presented, are all the underlying source data available to ensure full reproducibility?

Not applicable

Is the topic of the practical tips discussed accurately in the context of the current literature

Yes

Are all factual statements correct and adequately supported by citations?

Yes

Are the conclusions drawn balanced and justified on the basis of the presented arguments?

Partly

Are arguments sufficiently supported by evidence from the published literature and/or the authors’ practice?

Yes

Reviewer Expertise:

Online education, adult education, educational technology, faculty development

I confirm that I have read this submission and believe that I have an appropriate level of expertise to confirm that it is of an acceptable scientific standard, however I have significant reservations, as outlined above.

# Response

**Published:** 2024-07-03

**Authors:** Chloé Bras

**Affiliations:** FdG, Universiteit van Amsterdam, Amsterdam, North Holland, The Netherlands

Dear Anita Samual,  Thank you for reading and for your feedback. We have made changes to the paper and its uploaded as Version 2. We believe that version to is much better thanks to your review and feedback.  Below we indicate point by point how we have adjusted it in version 2: 

- *Introduction: In your Introduction, you say, The educational institute plays a major role in facilitation of the process. What ‘process’ are you referring to here? It is not clearly identified in the preceding sentences.*

We clarified this with: “transition to online education”

- *The Community of Inquiry framework was proposed and developed by Garrison and Archer in 1999. While Richardson et al. have used the framework, Garrison and Archer should be acknowledged.*
  We added Garrison and Archer as a reference and stated that the framework of Richardson et al. was based on the framework of Garrison et al.

 

- *You note that your tips are divided into 4 themes. It would be helpful to identify which theme aligns with which tip.*

Before the tip we added the theme it aligns to (faculty development, infrastructure, student engagement or teaching activities)  

- *Furthermore, how do these themes and tips align with the CoI model? This needs to be clearly stated.*

In every tip we clarified to which aspect of the CoI model it aligns.  

- *Tip 1 refers to a core educational team. It would be helpful to define what you mean by this. While the title mentions ‘collaboration,’ the tip itself primarily discusses communication. Clarification is needed here.*

We clarified core educational team as people who teach the most and added communication to the title.  

- *The title for Tip 2 refers to educational ICT infrastructure. However, you only discuss streaming technologies. Educational ICT is much broader than this. You might want to consider reframing your title for this tip.*

Thank you for your suggestion, we reframed the title of the tip to make the focus of the tip more clear.  

- *In paragraph 2 of Tip 3, you say “abovementioned tools.” What tools are you referring to?*

We were referring to the exam platform. We rewrote the sentence to make it more clear.  

- *Similar to tip 2, there is a disconnect between the title and description of Tip 3. The title of Tip 3 refers to digital tools. But the only tools you really discuss are assessment tools. While assessment tools can fall under digital tools, they are not the ONLY digital tools.*

Thanks for addressing, it’s true that there are more digital tools. We adjusted the title from digital tools to assessment tools as you suggested.  

- *Tip 4: In paragraph 2, you say, To enable everyone involved in online teaching to participate in faculty development activities, both formal and informal faculty development approaches should be used. Since the title for the tip mentions ‘formal’ initiatives, this sentence can be removed since the next tip is about informal initiatives.*

You are right, and it was also mentioned in Tip 5, so we removed this part in Tip 4.  

- *Tip 7: Paragraph 1 uses the phrase, during the same period. What period are you referring to here?*

We were referring to the period of online education. We adjusted the text to make it more clear.  

- *Tip 8: The word “stimulate” is used multiple times. It might help to use different words.*

We changed the text and used different words to make it more clear and easier to read.  

- *Tip 9: Paragraph 1 you use the phrase “the teacher streamed with video and audio.” I’m not sure what you mean here. Some clarification on what you mean by “streamed with video and audio” would be helpful.*

We meant that the teacher was continuously visible during the teaching session. We rephrased the sentence to make it more clear.
